# Vaccination status of resident pediatricians and the potential risk for their patients - a cross-sectional questionnaire study in pediatric practices in Vienna

**DOI:** 10.1186/s12887-019-1529-0

**Published:** 2019-05-16

**Authors:** Susanne C. Diesner, Sarah Peutlberger, Peter Voitl

**Affiliations:** 1 First Vienna Pediatric Medical Center, Donaucitystrasse 1, 1220 Wien, Austria; 2 Sigmund Freud University Vienna, Freudplatz 1, 1020 Vienna, Austria

**Keywords:** Vaccination status, Pediatricians, Vaccine preventable disease

## Abstract

**Background:**

Pediatricians are advised by the Austrian ministry of health to be vaccinated against diphtheria, tetanus, pertussis, poliomyelitis, measles, mumps, rubella, varicella, hepatitis A, hepatitis B, meningococcus ACWY and meningococcus B, pneumococcus and seasonal influenza. As they take care of a vulnerable patient group including newborns and infants, who have not been vaccinated yet, it is important that they have a positive immunization status in order to protect their patients. This cross-sectional study aims to investigate the vaccination status of pediatricians and their assistants in practices in Vienna.

**Methods:**

All 196 resident pediatricians in Vienna were invited to participate in this cross-sectional, questionnaire-based study. They had to specify their sex, medical profession, self-reported vaccination status for the respective vaccine preventable diseases and the type of practice they are working in (private versus government funded practice).

**Results:**

High vaccination rates above 90% were found for measles, poliomyelitis, pertussis and hepatitis B, whereas seasonal influenza, meningococcus and pneumococcus were the least accepted vaccinations in this cohort. No significant differences were observed for male and female vaccination habits. Influenza and pneumococcus vaccines were more frequently received by pediatricians than their assistants. Health care workers (HCW) of private practices had significantly lower hepatitis B vaccination rates compared to those working in practices covered by the Vienna health insurance fund.

**Conclusion:**

Resident pediatricians in Vienna reveal rather high vaccination rates for some but not all of the recommended immunizations, which puts their pediatric patients at risk. Measures for higher vaccination rates are needed especially for this medical professional group.

**Electronic supplementary material:**

The online version of this article (10.1186/s12887-019-1529-0) contains supplementary material, which is available to authorized users.

## Background

Health care workers (HCW) have an increased risk to acquire and transmit certain infectious diseases not only to colleagues but in particular to vulnerable patients, such as newborns and children [1, 2]. Vaccination of medical professionals reduces the risk of infections, nosocomial transmission and helps to maintain health care delivery in times of disease outbreaks [3], for instance during influenza season. The European Center for Disease Prevention and Control recognized health care professionals as most important source of vaccination information for the general population. But only when they are aware of vaccination plans and side effects, proper advice can be given to patients, which might help to overcome the phenomenon of the so-called vaccine hesitancy. The physician’s recommendations substantially influence the patients’ vaccination behavior making it obvious that the physician’s own vaccination habits might be a bias for their vaccination hesitancy [4]. Pediatricians are the first contact for parents and their children when questions regarding vaccination schedules have to be discussed. Pediatricians themselves should have proper vaccination coverage. Only then, newborns and young children, who are of particular risk to encounter vaccine preventable diseases due to their incomplete vaccination status might have some passive protection.

Due to these factors national policies are implemented for occupational vaccinations of HCW [5]. The Austrian ministry of health publishes an update of the vaccination recommendations every year. These vaccinations are, however, not mandatory, neither for the general population, nor for HCW. Austrian pediatricians are advised, but not legally bound, to vaccinate against diphtheria, tetanus, pertussis, poliomyelitis, measles, mumps, rubella, varicella, hepatitis A, hepatitis B, meningococcus ACWY and meningococcus B, pneumococcus and seasonal influenza [5].

Although these vaccinations are not mandatory by Austrian law, many hospitals and medical universities recently started to offer internships and positions only under the terms of positive immunization protection. The costs for these vaccinations are not reimbursed by the government, but some of the vaccines are offered by the employer for free or price-reduced. Thus, it might be speculated that HCW in Austria might reach a rather high vaccination coverage. That this is not the case at the moment is proven by a recent publication by Harrison et al., who found rather low immunization rates for certain vaccination preventable diseases in HCW of the Vienna General Hospital [6].

As far as we know there are no data available on the vaccination coverage of health care professionals working in pediatric outpatient clinics in Austria. Therefore, we conducted a cross-sectional questionnaire study on the vaccination rates of pediatricians and their assistants in all Viennese pediatric practices and analyzed the impact of gender, profession (pediatrician vs. assistant) and type of outpatient clinic (private practices in comparison to pediatric outpatient clinics covered by the Vienna regional health insurance fund).

## Methods

### Ethics approval

This cross-sectional study was conducted as anonymous questionnaire, which is the reason why an ethics approval was not necessary according to the “Ethics Committee of the city of Vienna” (Thomas-Klestil-Platz 8, 1030 Vienna, Austria, submission number: MA15-EK/17_162_VK_NZ). Participation was voluntary. All participants were explained the reasons and aims of this questionnaire and gave written consent by filling in the questionnaire.

### Questionnaire

The questionnaire (Additional file 1) included the following information: a) pediatrician / assistant, b) female / male, c) practice covered by the Vienna regional health insurance fund / private practice, d) contraindication against vaccination, e) immunization or protection due to former infection (e.g. measles) for measles, poliomyelitis, pertussis, influenza current season, influenza previous season, meningococcus B, meningococcus ACWY, pneumococcus and hepatitis B. Participants were asked to self-report their vaccination status according to their vaccination pass and according to the vaccination recommendations of the government.

We contacted all 196 pediatricians in outpatient clinics in Vienna who were asked to fill in the questionnaire. Their assistants also received a questionnaire. The questionnaire was designed without information on the participant’s name or date of birth and was returned anonymously into a letterbox.

### Inclusion and exclusion criteria

All Viennese resident pediatricians registered on the homepage of the medical association (www.praxisplan.at) were asked to participate in this study. Employed assistants, meaning logistic/nursing support to the pediatrician, but not medical trainees, working in a pediatric practice were included in this questionnaire study. There were no exclusion criteria.

### Statistics

Descriptive analysis of the data was done with IBM SPSS statistics, Microsoft Excel and Graph Pad Prism 6 software. Total numbers and frequency distribution were calculated. Fisher’s exact test was used in the analysis of contingency Tables. A *P* value < 0.05 was considered statistically significant.

## Results

### Study population

In total, 118 questionnaires were returned, of which 68 were answered by pediatricians (57.6%) and 50 by their assistants (42.4%). This corresponds to a response rate of 34.7% of all resident pediatricians in Vienna.

Independent on their profession, 33 questionnaires were completed by men (28%) and 79 by women (67%), six persons did not make a statement on their sex (5%). Thirty-five participants indicated working in a private practice (29.7%) compared to 73 HCW of government funded pediatric practices (61.9%). Ten questionnaires did not contain information on the type of practice (8.4%). Five of the 118 persons (4.23%) answered that they had a contraindication for one of the vaccinations, without providing any additional information on the cause.

### Vaccination rate

The vaccination rate, meaning immunity against the vaccine-preventable disease, was defined as either a history of natural infection (measles) or of completed vaccination. The recommended schedule includes basic immunizations either of 2 or 3 shots, or in case of diphtheria-tetanus-pertussis-poliomyelitis and hepatitis B a booster immunization every 10 years according to the national vaccination plan. Pneumococci vaccination for HCW is recommended to include the 13-valent conjugate followed by a booster vaccination with the 23-valent polysaccharide. The highest immunization rate of all study participants (Table 1) was found for measles (99.2%), followed by poliomyelitis (98.3%), pertussis (95.8%) and hepatitis B (93.2%). The recommended seasonal influenza vaccine was received by 72.9% of the respondents in the previous season and by 69.5% in the current season. Low vaccination coverage was observed for pneumococcus (46.6%), meningococcus ACWY (49.2%) and meningococcus B (32.2%); all vaccinations that are clearly recommended for all HCW of the pediatric field. Some of the participants did not specify their immunization status for all vaccine preventable diseases, indicated as “not specified” in Table 1.

**Table 1:** Vaccination coverage of health care workers at pediatric practices in Vienna

Vaccine preventable disease	Vaccinated		Not Vaccinated		Not specified	
	Percentage (%)	Absolute number (n)	Percentage (%)	Absolute number (n)	Percentage (%)	Absoute number (n)
Measles	99.2	117	0.8	1	0	0
Poliomyelitis	98.3	116	1.7	2	0	0
Pertussis	95.8	113	2.5	3	1.7	2
Influenza 2016/17	72.9	86	22.9	27	4.2	5
Influenza 2017/18	69.5	82	27.1	32	3.4	4
Meningococcus B	32.2	38	64.4	76	3.4	4
Meningococcus ACWY	49.2	58	50	59	0.8	1
Pneumococcus	46.6	55	50.8	60	2.5	3
Hepatitis B	93.2	110	4.2	5	2.5	3

### Gender differences in immunization status

Due to the fact that 6 participants did not indicate their sex, they were excluded from this sub-analysis, resulting in 79 female and 33 male participants. Of these 112, some were not able to specify their immunization status for all vaccine preventable diseases (indicated in Table 2). No significant differences were observed between male and female participants with regard to the specific vaccine preventable diseases. Some differences, although not statistically significant, were observed for influenza in the season 2016/2017, where only 68% of women were vaccinated compared to 82% of men.

**Table 2:** Immunization status for vaccine preventable diseases in female and male HCW

Vaccine preventable disease	Imunization status	Female (*n* = 79)		Male (*n* = 33)	
		Percentage (%)	Absolute number (n)	Percentage (%)	Absolute number (n)
Measles	Positive	98.7	78	100	33
	Negative	1.3	1	0	0
	Not specified	0	0	0	0
Poliomyelitis	Positive	97.5	77	100	33
	Negative	2.5	2	0	0
	Not specified	0	0	0	0
Pertussis	Positive	96.2	76	93.9	31
	Negative	2.5	2	3	1
	Not specified	1.3	1	3	1
Influenza 2016/17	Positive	68.4	54	81.8	27
	Negative	25.3	20	18.2	6
	Not specified	6.3	5	0	0
Influenza 2017/18	Positive	68.4	54	69.7	23
	Negative	30.3	24	21.2	7
	Not specified	1.3	1	9.1	3
Meningococcus B	Positive	29.1	23	36.4	12
	Negative	67.1	53	60.6	20
	Not specified	3.8	3	3	1
Meningococcus ACWY	Positive	46.8	37	48.5	16
	Negative	51.9	41	51.5	17
	Not specified	1.3	1	0	0
Pneumococcus	Positive	44.3	35	54.5	18
	Negative	53.2	42	45.5	15
	Not specified	2.5	2	0	0
Hepatitis B	Positive	92.4	73	97	32
	Negative	5.1	4	3	1
	Not specified	2.5	2	0	0

### Immunization status of physicians and assistants

Vaccination rates were significantly different between physicians and assistants for influenza in the season 2016/2017 (*P* = 0.044, 81% of pediatricians vs. 62% of assistants), which however was not prominent anymore in the following season 2017/2018 (*P* = 0.141). A trend towards significance was detected for the pneumococcus vaccine (*P* = 0.059), where up to 54% of physicians were immunized compared to 36% of assistants. All other immunization rates were evenly distributed among physicians and assistants (Table 3).

**Table 3:** Immunization rates of pediatricians and assistants in Viennese outpatient clinics

Vaccine preventable disease	Immunization status	Pediatricians (*n* = 68)		Assistants (*n* = 50)	
		Percentage (%)	Absolute number (n)	Percentage (%)	Absolute number (n)
Measles	Positive	100	68	98	49
	Negative	0	0	2	1
	Not specified	0	0	0	0
Poliomyelitis	Positive	98.5	67	98	49
	Negative	1.5	1	2	1
	Not specified	0	0	0	0
Pertussis	Positive	95.6	65	96	48
	Negative	2.9	2	2	1
	Not specified	1.5	1	2	1
Influenza 2016/17	Positive	80.9	55	62	31
	Negative	16.2	11	32	16
	Not specified	2.9	2	6	3
Influenza 2017/18	Positive	73.5	50	64	32
	Negative	20.6	14	36	18
	Not specified	5.9	4	0	0
Meningococcus B	Positive	38.2	26	24	12
	Negative	60.3	41	70	35
	Not specified	1.5	1	6	3
Meningococcus ACWY	Positive	54.4	37	42	21
	Negative	45.6	31	56	28
	Not specified	0	0	2	1
Pneumococcus	Positive	54.4	37	36	18
	Negative	42.6	29	62	31
	Not specified	2.9	2	2	1
Hepatitis B	Positive	95.6	65	90	45
	Negative	2.9	2	6	3
	Not specified	1.5	1	4	2

### Vaccination coverage in private practices in comparison to pediatric outpatient clinics covered by the Vienna regional health insurance fund

We compared the results of all HCW, meaning pediatricians and assistants, of private practices with those of pediatric practices covered by national health insurance funds. Eight participants did not specify the kind of practice they were working in and were therefore not included in this analysis. We observed a significantly higher immunization rate for hepatitis B in the personnel working in pediatric outpatient clinics covered by the health insurance funds (97%) compared to private practices (83%), (*P* = 0.031). All other immunization rates did not reveal any significant differences (Table 4).

**Table 4:** Immunization status of HCW working in private practices compared to pediatric outpatient clinics covered by the health insurance fund

Vaccine preventable disease	Immunization status	Government funded pediatric practices (*n* = 75)		Private practice (*n* = 35)	
		Percentage (%)	Absolute number (n)	Percentage (%)	Absolute number (n)
Measles	Positive	100	75	97.1	34
	Negative	0	0	2.9	1
	Not specified	0	0	0	0
Poliomyelitis	Positive	98.7	74	97.1	34
	Negative	1.3	1	2.9	1
	Not specified	0	0	0	0
Pertussis	Positive	94.7	71	97.1	34
	Negative	2.7	2	2.9	1
	Not specified	2.7	2	0	0
Influenza 2016/17	Positive	74.7	56	65.7	23
	Negative	20	15	31.4	11
	Not specified	5.3	4	2.9	1
Influenza 2017/18	Positive	69.3	52	65.7	23
	Negative	26.7	20	31.4	11
	Not specified	4	3	2.9	1
Meningococcus B	Positive	32	24	31.4	11
	Negative	62.7	47	68.6	24
	Not specified	5.3	4	0	0
Meningococcus ACWY	Positive	49.3	37	45.7	16
	Negative	49.3	37	54.3	19
	Not specified	1.3	1	0	0
Pneumococcus	Positive	50.7	38	42.9	15
	Negative	48	36	54.3	19
	Not specified	1.3	1	2.9	1
Hepatitis B	Positive	97.3	73	82.9	29
	Negative	1.3	1	11.4	4
	Not specified	1.3	1	5.7	2

## Discussion

Pediatricians are at higher risk to encounter vaccine preventable diseases in comparison to other medical professions due to the vulnerable patient group they are taking care of. Therefore, they are advised to be immunized against these diseases before starting their career in this field. The vaccines recommended for all HCW in pediatric clinics in Austria include diphtheria, tetanus, pertussis, poliomyelitis, measles, mumps, rubella, varicella, hepatitis A, hepatitis B, meningococcus ACWY and meningococcus B, pneumococcus and seasonal influenza [5]. However, we observed a rather broad range of vaccination rates for the different vaccines in our study. Measles, poliomyelitis and pertussis reached an immunization coverage above 95% representing the needed vaccination coverage for eradication of measles according to the World Health Organization [7], which however is not reached in the general population in Austria underlined by the fact that 1 out of 3 15- to 30-year-olds lack the second measles vaccine [8]. When these results are compared to a recent publication of HCW in the general hospital of Vienna substantial differences were identified. In line with our results Harrison et al. found high vaccination rates for poliomyelitis (93%) but rather low immunization protection for measles (59.8%) and pertussis (58.2%) [6]. Hepatitis B vaccination was similar in both studies (93%). It might be expected that HCW in the same city, Vienna, should have comparable vaccination habits. One of the reasons for this discrepancy might be the participating cohort and the small sample size, being one of the major limitations of this study. Only 34.7% of all resident pediatricians returned the questionnaire compared to 58% of HCW in the study be Harrison et al. [6]. It might be speculated that those pediatricians who follow a “pro-vaccination” attitude were more willing to answer the questionnaire, while other physicians who are more critical towards vaccine recommendations refused to participate. We intended to avoid this bias by ensuring anonymity to all participants but we cannot rule out some discrepancy.

In international comparison, the vaccination rates of the Viennese resident pediatricians score well. In France, a similar study was conducted among general physicians from the Loire region. Eighty-one percent of respondents stated to be immunized against poliomyelitis, 59% against pertussis, 73% against influenza, 87% against hepatitis B and 64% against measles [9], while Greece data reported on a completed vaccination rate being rather low for measles (33% of HCW) and even lower for hepatitis A (5.8%). Interestingly, in this cohort, vaccination coverage was indirectly associated with age, as younger HCW received more vaccines than older [10]. Hepatitis B vaccination is quite well accepted by HCW reaching an immunization rate between 75 and 100% according to the Venice II project report [11], whereas HCW are much less willing to immunize against measles-mumps-rubella, which reached only 9.7% vaccination coverage in an Italian study [3]. Data on the vaccination rates for meningococcus and pneumococcus are rare. In comparison to the results of Harrison et al. our data revealed a rather high vaccination coverage (32 to 49% respectively in comparison to 6.7%), which might be originated in the national recommendations in particular for pediatricians to receive these vaccines.

With regard to vaccination habits of medical personnel we identified differences in one of the investigated influenza seasons where significantly less assistants were immunized in comparison to pediatricians. This was also prominent for the pneumococcus vaccine. As assistants have close contact to pediatric patients they represent a potential source for transmission of these pathogens especially during winter season, when both pathogens are commonly present and put in particular newborns and infants at risk. Influenza vaccine is one of the least accepted vaccinations among medical professionals. In a US study, HCW in hospitals were immunized against influenza more often than resident physicians [12]. This discrepancy was also prominent in our study with 70% of participants being vaccinated against influenza in comparison to 42% of Viennese health care professionals in the general hospital [6]. The latter data are comparable to a Greece study, where 45.9% of HCW had a history of influenza vaccination at least once [10], whereas in a Canadian study, even 69 to 76% of residents were vaccinated against influenza [13]. In contrast, in the general population in Austria the influenza vaccination coverage is only around 5%. Thus, it is obvious that the vaccination coverage is far from the target of 75% to be achieved as stated in the EU council recommendation of 2009 [14]. Pertussis vaccination follows a similar pattern. In Germany, 48.2% of university doctors but only 13.4% of nurses were vaccinated against pertussis in the past 10 years [15]. Many of the HCW do not even know whether they received the vaccine, which might be due to its part as a combination vaccine with tetanus, poliomyelitis and diphtheria. This puts the patients at risk to encounter these bacteria, which is in particular dangerous for infants and newborns who are not protected by maternal antibodies and have not been vaccinated yet. A rise in pertussis cases was described in Austria in the recent years [16] underlining the importance of a broad vaccination coverage.

One of the trends in Austria is to have their children checked by pediatricians working in private practices and not in government funded practices. Therefore, we were interested if the vaccination coverage of these two pediatric clinic types show comparable results. Only the immunization protection for hepatitis B was significantly lower in HCW of private practices. The reason for this phenomenon remains unclear and cannot be explained by this study.

The strength of our study is that it provides an insight into the vaccination habits of HCW in the pediatric field in Vienna. Making these data public might lead to an awareness of pediatricians not only in Vienna to get their vaccination status up to date. However, the study design is one of the limitations, as the data are only self-reported without any control of the actual vaccination documentation, which might lead to a bias of results. The reason for the rather low reply rate remains unclear. We guaranteed all participants anonymity but it might reflect the low value that vaccinations have for some HCW.

## Conclusion

Based on the results of this and other studies, it is obvious that HCW especially of the pediatric field do not follow international vaccination recommendations for all vaccines. While rather high vaccination rates are observed for measles, poliomyelitis and pertussis in Viennese resident pediatricians and their assistants, important vaccines, such as meningococcus and pneumococcus are more neglected by HCW. This might be due to a lack of knowledge that these vaccines are recommended. A nationwide campaign informing all pediatricians and their employees would improve the knowledge and might affect their vaccination habits. The main reason for missing vaccinations is the lack of active offer for vaccination [17]. If vaccines are offered free of charge to all HCW of pediatric practices and if employers make a positive immunization protection a condition of employment pediatricians might be efficiently motivated for checking their vaccination status and in case of deficiencies might catch up the recommended vaccines.

## Additional file

Additional file 1: Vaccination status of resident pediatricians and the potential risk for their patients. (DOCX 14 kb)

## Abbreviations

HCW: Health care workers
MA15: Magistratsabteilung 15 of the city of Vienna
n: Number
