# Supplementary material for: Vaccination status of resident pediatricians and the potential risk for their patients - a cross-sectional questionnaire study in pediatric practices in Vienna
Source: BMC Pediatr. 2019 May 16;19:153. doi: 10.1186/s12887-019-1529-0 (PMC6521505; doi:10.1186/s12887-019-1529-0)
Supplement: Supplementary file 1 — Vaccination status of resident pediatricians and the potential risk for their patients. (DOCX 14 kb) [file 12887_2019_1529_MOESM1_ESM.docx]

**QUESTIONNAIRE**

**“Vaccination status of resident pediatricians and the potential risk for their patients”**

**Medical Profession:**

- Pediatrician
- Assistant

**Sex:**

- Female
- Male

**Type of practice:**

- Private practice
- Government funded practice

**Do you have any contraindication against vaccination?:**

- yes what kind of contraindication? _________________
- No

I have immunity / protection against the following vaccine-preventable diseases / pathogens (either due to complete vaccination according to the national vaccination recommendation or to former natural infection (measles)):

| **Vaccine preventable disease** | **YES** | **NO** |
| --- | --- | --- |
| Measles |  |  |
| Poliomyelitis |  |  |
| Pertussis |  |  |
| Influenza |  |  |
| - Previous season |  |  |
| - This season |  |  |
| Meningococci B |  |  |
| Meningococci ACWY |  |  |
| Pneumococci |  |  |
| Hepatitis B |  |  |
